# An Evaluation of the Acceptability, Appropriateness, and Utility of a Bibliotherapy for Children of Parents With a Mental Illness

**DOI:** 10.3389/fpsyt.2022.815873

**Published:** 2022-04-14

**Authors:** Kelly Vetri, Geneviève Piché, Aude Villatte

**Affiliations:** ^1^Laboratory LaPProche, Department of Psychoeducation and Psychology, Université du Québec en Outaouais, Saint-Jérôme, QC, Canada; ^2^Centre de recherche universitaire sur les jeunes et les familles (CRUJEF), Québec, QC, Canada; ^3^Réseau de recherche en santé des populations du Québec (RRSPQ), Montréal, QC, Canada; ^4^Réseau Intersectoriel de Recherche en Santé de l'Université du Québec (RISUQ), Québec, QC, Canada

**Keywords:** children, parental mental illness, bibliotherapy, psychoeducation, interpersonal psychotherapy, intervention

## Abstract

**Background:**

Children of parents with a mental illness are at higher risk for various psychiatric problems and adaptive difficulties compared to those of parents without mental health problems. Certain preventive psychoeducational interventions target these children to promote their well-being and resilience and prevent the emergence of adaptive difficulties. However, few such interventions have been developed and evaluated specifically for elementary school-aged children of parents with a mental illness.

**Objectives:**

This study aims to evaluate an interpersonal psychotherapy-based book targeting children living with a parent with a mental illness.

**Methods:**

The study examines children, parents and psychosocial workers' perception of the acceptability, appropriateness and utility of the book. In total, 22 participants answered online open-ended questions after reading the book.

**Results:**

The book was highly appreciated and positively perceived by the families and psychosocial workers. Results suggest that children, parents and psychosocial workers viewed it as an appropriate and useful tool for supporting children with a parent with a mental illness. The present study reveals that the bibliotherapy appears well adapted to the developmental level of school-age children.

**Discussion:**

This study presents a book that shows promise for supporting the resilience of elementary school-aged children having a parent with a mental illness. Results highlight the importance of tailoring the content and modalities of interventions to the developmental level, needs and preferences of elementary school-aged children. The relevance of a collaborative method is also discussed, thus providing knowledge regarding this type of approach for the development of interventions targeting children.

## Introduction

Overall, about one in five children have a parent with a mental illness (1). The mental illness symptoms can have a significant impact on the parent and other family members, including children (2). Living with a parent with a mental illness may involve adverse family conditions (3, 4) characterized by high levels of conflict, poor family communication and low cohesion between family members (5, 6). Children of parents with a mental illness (COPMI) frequently report a poor parent-child relationship, few moments of shared family time, a tendency to be parentified (7) and, above all, developmental needs that often go unmet (1). Exposure to parental mental illness can also lead to several negative outcomes for a child. COPMI are at higher risk of a wide range of negative affects (e.g., fear, anxiety, sadness, guilt, shame, confusion), notably owing to the parent's symptoms, lack of information about the mental illness, and fear of developing a similar illness or being responsible for the parent's illness (8, 9). These children may also experience more isolation and loneliness than others (10). The stigma of their parent's mental illness may make them reluctant to invite friends over, talk about the mental illness, or ask for help from those around them, thus reinforcing their feelings of distress (10–12). Moreover, COPMI are at higher risk of academic difficulties (8, 13, 14) and emotional and behavioral problems (15, 16). The scientific literature reveals, furthermore, that parental mental illness significantly increases a child's risk of developing psychopathology during childhood, adolescence and well into adulthood (17–22). This risk of intergenerational transmission seems present for two future generations (22). Furthermore, the likelihood of these children developing a mental illness at a younger age is higher than that observed in children in the population at large (23, 24).

Despite these important risks, it should be noted that children exposed to parental mental illness do not all develop in the same manner, as is underlined by the multifinality concept. While some face significant psychosocial difficulties, others demonstrate resilience in the face of family adversity and avoid developing psychosocial problems (1, 25–28). Interestingly, according to a literature review by Drost et al. (25), several COPMI even report feeling “*more mature, independent, and empathic than their peers who did not have a parent with a mental illness*”. These resilient children show that some aspects of their environment and family, including positive and strong social support or the presence of a parent with positive mental health, helped them maintain good mental health. At the same time, the literature indicates that interventions to promote the mental health, well-being and resilience of youth with a mentally ill parent should include several components underscoring protective psychosocial factors. Protective factors are: seeking and requesting social support; acquiring knowledge about the parent's mental illness; communication and problem-solving skills; self-esteem and social competence skills (e.g., recognizing and sharing feelings and needs); and capacity to replace negative cognitions (12, 29, 30). Coping skills are particularly important in a family context where the manifestations of parental mental illness can lead parents to be unable to manage daily problems and conflicts.

Generally speaking, results of several studies indicate that the preventive interventions designed for this at-risk clientele, including the targets mentioned above, are beneficial and have positive effects on children's mental health and overall well-being (9, 31–33). They are recommended as early as elementary school, in a prevention perspective (1, 5, 30, 34). As examples, the *Play and Talk* group program (21), targets 8-to-12-year-old children with a mentally ill parent and offers eight weekly 90 min group meetings and a family meeting. The intervention program shows beneficial effects in participating families. Indeed, in a study conducted with 254 families, a decrease in negative thoughts and emotional and behavioral problems was observed in young participants to this program compared to the control group (35). Another example of an intervention with beneficial effects is the *Family Focus DVD*, designed for families with a parent having a depressive or anxiety disorder and elementary school-aged children. An increase in mental health literacy and knowledge of strategies for talking about mental health problems was reported by the 29 young participants (36). More generally, a meta-analysis highlighted that COPMI have a 40% lower risk of developing psychopathology when they have access to effective preventive services (16). Therefore, it's essential to support COPMI and their families with effective and easily accessible interventions to help them cope with the various challenges they face (37). The challenges they face are exacerbated by the current epidemiological situation and the difficulty of the mental health services to respond quickly and in an adapted way to their important needs (38–40).

However, even if the effects of interventions are positive and seem to persist and even increase over time, they tend to be small and are therefore unlikely to be sufficient to effectively reduce these children's risk factors or lighten their burden (35, 41–43). Some researchers raise the hypothesis that the content and modalities of certain preventive programs and tools for elementary school children may not be adequately tailored to their needs, characteristics or developmental level (35, 41, 42). In fact, very few published studies present information on the satisfaction or perceived usefulness of their content or intervention modalities by participants or on how the program content is integrated into the children's and family's life (44, 45). Besides, few studies have consulted elementary school-aged children to determine their needs and preferences when designing or evaluating interventions for them (44). Hence, almost no information is available on how COPMI perceive and accept the tools and programs proposed to them or, for example, if they understand the terms, strategies or activities included (42, 44–46). In general, the effects of preventive interventions are usually evaluated without considering their implementation (47). The adequacy between the characteristics of the clientele, the intervention protocols and the service delivery can ensure successful implementation and utilization of available services among families (48–51). When a psychoeducational program or intervention tool is offered for school-aged children, the proposed content must be relevant and adapted to their developmental level, notably their ability to understand complex concepts, solve interpersonal problems, focus during long periods of time, and recognize and share their emotions. Garber et al. (42) suggest these factors may modulate the implementation and effect sizes of psychoeducational programs or interventions targeting children.

Some strategies could improve children's acceptability and understanding of the content of an intervention. The recommendation is to use simple language and clear and short sentences when giving instructions, and to provide tangible and visual materials, pictograms, playful games, concrete examples and practical experiences (42, 52). Bibliotherapy appears to be among the most promising ways to promote the understanding of psychoeducational content and its adaptation to children's developmental level (52–54). The term *bibliotherapy* refers to the use of written materials (e.g., storybooks) as therapeutic tools for preventing or treating a variety of specific issues related to a child's needs (54, 55). Empirical evidence underlines the positive effects of bibliotherapy on individuals with mental health problems and on mental health and well-being in general (55, 56). Also, although the evidence is scarce, some evidence tends to show that bibliotherapy could be beneficial for COPMI (57–59). In fact, some interventions for COPMI incorporate psychoeducation and informative materials in written form (e.g., pamphlets, books, symbolic stories), which supports the relevance of this type of medium for these children (30, 33, 60).

In Quebec, Canada, a book titled *Le Trésor de l'Île Rouge* (see https://lapproche.uqo.ca/le-tresor-de-lile-rouge/) was developed initially as part of a preventive intervention program for families with a depressed parent adapted from *Family Talk* (61), *FAMILLE*+ (62, 63). The aim of the book is to help elementary-school COPMI learn coping strategies to deal with the challenges and difficult situations of everyday life. The book can be read by the child alone or facilitated by a professional, parent or other adult. The story describes an amusing pirate adventure where children are introduced to the main characters, their adventures and the strategies they use to overcome the obstacles and challenges they encounter. It uses different learning modalities and a series of activities offering young readers various opportunities to learn while having fun. Some of these are free to download and use on our website (e.g., Emotional-Pirate tools). The book's content is based on intervention targets acknowledged to be most relevant to COPMI as presented above (e.g., (19, 29, 64). It is also based on the Interpersonal Psychotherapy (IPT) approach, a commonly used and evidence-based preventive intervention treatment for depression in adolescents (65, 66) and pre-adolescents (8–12 years old; (67)) and a number of other disorders including anxiety (68). Several studies show its effectiveness for improving well-being and social functioning in young people (66). IPT focuses on improving interpersonal relationships, reducing symptoms of mental illness and improving well-being. This goal is achieved by improving communication and relational problem-solving skills and promoting positive interpersonal behaviors with significant others (e.g., parents, peers). Similarly, our objective via the IPT-based book is to help elementary school-aged COPMI learn concrete socio-emotional regulation skills, effective communication and interpersonal problem-solving skills, strategies for reducing conflict and ways to promote their social support network. In the long term, this may enable children dealing with parental mental illness to better cope with their parents and family. It may also help promote a positive parent-child relationship, children's well-being and, ultimately, their mental health (see Table 1).

**Table 1:** Strategies and activities of the book.

**Chapter Title**	**Description and activities**	**Psychoeducational goals** **and strategies**
Captain Philou and his new crew	Captain Philou puts together his new crew to find the Red Island Treasure and the crew members use all their strengths to battle enemy pirates.	Use your strengths (personal and family).
The storm rages on	The crew must overcome a terrible storm, which makes the pirates experience a lot of emotions and jeopardizes the operation of the ship.	Share your emotions with others, otherwise they will grow inside you.
The island of a thousand words	Poor communication between the crew members causes the boat to crash on an island.	Use your communication skills. Work together to overcome challenges.
The rotten fish	The captain is not feeling well. A pirate must take on his responsibilities. He has too many tasks and the ship is attacked by sharks.	Ask for help when you need it. Recognise and talk about your limits and needs.
The red ants	The pirates arrive on the Red Island. Excitement is running high, and pirates experience all kinds of emotions. They make a plan to overcome the obstacles they will encounter on their way.	Breathe gently through your belly to calm yourself. Use the problem-solving technique.
A celebration	The crew hikes over the Red Island and faces multiple dangers. They find the brother of a teammate and the treasure. A banquet is held to celebrate.	Emphasize the importance of doing fun activities as a family.
Captain's questions	Reading comprehension questions: 5 multiple choice questions related to the story and strategies taught.	Validation and learning integration of coping strategies.
Pirate-Active	Body percussion activities: Pirate dances and related reflection questions.	Get the children moving and make connections to the theoretical content.
Emotional-Pirate	Feelings tools: Mood thermometer and emotion display to evaluate feelings.	Encourage identification of emotions and generate discussion on the subject.
Tips from Diane La Sage	Emotional regulation skills: 3 tips for sharing and calming your emotions. Space to write down ideas.	Encourage sharing of emotions and healthy strategies to soothe emotions.
Quiz: Which pirate are you most like?	Identifying personal strengths: Quiz with questions and captions to determine which pirate child resembles.	Explore and reflect on personal strengths.
Special recipes from Jeanne La Borgne	Parent-child activities: 4 recipes related to the story about the pirates.	Promote quality time between the child and a significant adult.
My pirate team	Social support activity: 1 proximity circle exercise and 1 fun pirate drawing activity.	Identify people who can meet the child's needs.
The pirate apprentices' corner	Self-esteem drawing activity: Invitation to draw the crew's adventures with the chance to post the drawing on the book's website.	Promote children's self-esteem and their integration of information and reinforce the dynamic aspect of the book.

It should be noted that the book addresses the main problems encountered by COPMI without addressing the specific issue of parental mental illness. Communication difficulties, impaired family functioning and a tendency to parentification are examples of themes highlighted in the characters' stories. For example, the main character, the ship's captain, feels unwell and can no longer meet the needs of his shipmates. This passage conjures the mental illness of a parent who has difficulty responding adequately to their child's needs. The captain also experiences many negative emotions (e.g., shame or guilt) and hides in his room rather than inform the crew of his situation. The captain is concerned about the possible reaction of the ship's company to his inability to function as usual, a situation recalling the stigma and shame associated with parental mental illness in families (10, 69).

Two previous pilot studies assessed the evaluation of the first version of the book and highlighted suggestions for improvement (70). Some changes have been made to enhance its acceptability for children ages 7 to 11. This study evaluates the second version of this book. The present study aims to evaluate the IPT-based book *Le Trésor de l'Île Rouge* targeting elementary school-aged children with a parent with a mental illness.

## Methodology

### Research Design

The formative evaluation examined children, parents and psychosocial workers perception of the acceptability, appropriateness and utility of the book. Qualitative data were collected by asking participants to respond to online open-ended questions after reading the book. Data triangulation between the perspectives gathered from children, parents and psychosocial workers was performed as a cross-validation modality to increase the validity and confidence level of research results (71). Involving children themselves in the assessment process is advocated as it provides more valid and accurate data based on their perspectives and experiences, which are often different from their parents or psychosocial workers (72–77).

### Recruitment and Procedures

Between August 2020 and September 2021, participants were recruited from two community organizations (*La Boussole* and *L'Apogée*) that provide help and support services for the relatives of individuals with a mental illness. The organisations' management promoted the research to families using their services and to psychosocial workers. To be eligible, families had to have a child aged 7 to 11 years old and a parent with a mental illness. For psychosocial workers, the single inclusion criterion was to work with families with parental mental illness. Participants had 3 weeks to read the book and complete the online confidential formative evaluation questionnaire. Although parents could help their child technically (e.g., write down the answers or rephrase the questions), they were told it was important for children to give their own answers when completing the questionnaire. A research assistant offered to assist children and parents as needed, but this help was not requested. Participating families were given a financial compensation of $25 in the form of a voucher.

### Sample

In total, 22 participants completed the online questionnaire, including eight parents (five mothers and three fathers), eight children (four girls and four boys) and six psychosocial workers. The children's explicit consent was obtained as part of their participation. Three children were 7–8 years old; two children are 9–10 years old, and three children were eleven years old (*M* = 9 years). The participating parents were aged between 36 and 45 years old (*M* = 40 years). Four families were biparental, two were blended or uniparental. Two children had an attention deficit hyperactivity disorder and were being treated by a child psychiatrist. All participating children were receiving specific support services from a community organization for the family members of a person with a mental illness. Four parents had a diagnosis—mainly of mood or anxiety disorders—and four were the partner of a parent with a diagnosis. All four parents with a diagnosis had a comorbid mental illness with one or more co-occurring illnesses. Five parents had a university degree, two had a college degree and one parent reported having a high school diploma. All six psychosocial workers were woman. Four of them were under 35 years and two were between 40 to 45 years old. Five had a university degree and one indicated having a college degree. The majority had one to 2 years of experience in their workplace.

### Formative Evaluation Questionnaire

To gather information concerning the book's acceptability, appropriateness and utility for children aged 7 to 11 years old with parent with a mental illness, participating children, parents, and psychosocial workers were asked open-ended questions about their overall appreciation of the book and its features. Examples of questions for children are the following: “Did you enjoy the book? Why?”; “What did you like most and least?”; “What strategy did you find most useful? Why?”. Most of the questions asked to the parents and psychosocial workers were identical. For examples: “Did you enjoy the book?”; “Do you think this book was helpful and useful?”; “Would you recommend this story to families with school-aged children?”. Psychosocial workers were asked one additional specific question: “Would you like to use this tool in your practice with children aged 7–11 and their families?”. Parents were also questioned about their perception of their child's reading experience, with one question: “In your opinion, did your child like the book?”.

Then, four specific open-ended questions were asked to psychosocial workers concerning the content's readability and appropriateness to the children's age and developmental level: “Do you think this book is adapted to the developmental capacities of the target clientele?”; “Do you think some aspects are difficult for children this age to understand?”; “Generally speaking, do you think the information is well explained?”; “In your opinion, is this book fun and enjoyable to read for children of this age group?.” Similarly, parents were invited to indicate in a few lines whether they considered the book to be appropriate for children the same age as their own and to justify their answers.

### Analysis Strategies

The qualitative data obtained have been transcribed and were processed using N'Vivo software (78). General categories were created to group the data according to the questions asked and the book's features. A triangulation of the data obtained from all respondents was also carried out (79). Excerpts of responses were selected for a concrete illustration of each category. Participants were assigned fictitious names. Recurrences or discrepancies in the responses of the various participants (parents, children, psychosocial workers) were highlighted.

## Results

### Evaluation of the Book in General

All children, parents and psychosocial workers who participated in the study reported they liked the book. More specifically, all parents affirmed their children appreciated it, as testified by this father of a 7-year-old girl: “*My daughter really liked the book. When she started reading, she would take it with her in the car, even when we went to the grocery store. Which actually surprised me because she's hardly ever done that with other books. She practically finished reading the book before I did.”* Furthermore, participating parents reported that the book was a helpful and useful tool for their child and family. Among the most relevant elements underlined, they mentioned accessible content, problem-solving strategies and understanding of experienced emotions, as the following examples demonstrate:

“*It makes them see that sometimes there are difficulties, but that it's always possible to overcome them together*.” (Anna, Mother of an 8-year-old girl)

“*The children realised that with good strategies they could solve their problems easily*.” (Jade, Mother of an 8-year-old girl and an 11- year-old boy)

“*In 2021, I think boys need to keep up with the times and this is a great way to show them that they can be close to their emotions even if they want to be strong all the time*.” (Layla, Mother of two boys aged 8 and 10)

The psychosocial workers all agreed the book was helpful and useful for 7- to 11-year-old children and their families. The two main reasons for the consensus were teaching strategies and use of the story as a therapeutic tool. In this regard, psychosocial workers reported that the book allowed children to access varied, concrete and simple tools. In their opinion, the proposed strategies are relevant to any child in the 7-to-11 age group and can be applied in a variety of situations to help them overcome difficulties. A few psychosocial workers and one parent also reported that using a story to address sensitive topics (e.g., difficulties or emotions) and teach positive coping strategies can improve children's attention, illustrate content and offer a playful setting for learning and expressing experiences. The following are some of their observations:

“*This puts the strategies one must use to be a self-benevolent and emotionally attentive human being into a more amusing context*.” (Layla, Mother of two boys ages 8 and 10)

“*I think this book has great potential for youth intervention. (…) I believe this book is helpful for any 7- to 11-year-old child because it offers strategies for communication, emotional regulation and self-management that may be relevant to them in the future. It also gives them the tools to remain calm in difficult situations. (...) The fact is that children can identify a situation in the book and receive a tool to solve it directly in the story. They can then apply it to themselves afterwards.”* (Florence, psychosocial worker)

Thus, all psychosocial workers agreed they would recommend the book to families with school-aged children and aimed to use it in their clinical practice with this clientele.

### Evaluation of the Book's Features

Families and psychosocial workers were asked to comment on their appreciation of specific features (e.g., story, complementary activities, characters, illustrations) of the book. The following sub-sections describe the perspectives of participants regarding the book's features.

#### Story and Characters

In general, all the families and most of the psychosocial workers reported they appreciated the story. The participants considered it interesting, funny, captivating and imaginative. Jess (psychosocial worker) explained: “*There are many twists and turns, and the adventure style is appealing.”*

Parents especially appreciated the parallels between the situations in the story and those in family life, as the following excerpts show: “*Nice parallel to life in general. I liked that it shows both the good times and the not-so-good times since these are a part of family life*.” (Evelyne, Mother of 11-year-old girl); “*Somehow, I saw myself in the role of Captain Philou. The book uses fantasy to teach life values that I think I and my children need. In fact, I asked my 13-year-old son to read it too.”* (Donovan, Father of 7-year-old girl). This father also praised the way the six chapters combined theory and practice “*in a very simple and understandable way*”.

Finally, one psychosocial worker seemed less appreciative of the story, which she deemed overly literal and redundant: “*I didn't feel there was a narrative, but rather a snapshot of each event (e.g., a challenge arises, you find solutions, you apply a solution, it works, you move on to the next challenge). It would have been interesting to have a larger narrative framework, which would create better flow in the story and smoother interactions between the characters*.” (Valerie, psychosocial worker)

In another vein, many children, psychosocial workers and parents found the characters to be well described, diverse, colorful, original, and interesting. Mathieu, a father of two children 8 and 9 specified: “*The characters are endearing and relatable to children.”* One social worker, on the other hand, commented that the characters were presented in an idealized fashion (e.g., the story failed to mention their bad habits, problems or faults). Another suggested that more connections could have been made between the reactions of the characters and mental health issues.

#### Illustrations, Visuals and Format

Children, parents and psychosocial workers reported liking the visuals, colors and illustrations of the book. These features are eye-catching and help make the book more dynamic. A psychosocial worker also noted that the color-highlighted tips and tools make it easy to identify the strategy used in the particular situation. Florence (psychosocial worker) explained: “*I think the book is super well done. The illustrations are very beautiful. (...) I really like the choice of colours.”*

Most children, parents and psychosocial workers really liked the book's format. Psychosocial workers specifically liked the division of chapters and sections and the balance between text and drawings, which made the book easy and pleasant to read and allowed for reading one chapter per day. However, two felt it contained too much information (e.g., a lot of details on the instruction page) and was “*a little too long and busy*”. As well, two said the activities should be presented after each chapter related to them, while another suggested the exercises be detachable so children could use them more readily.

#### Strategies Presented

When asked about the most valued features of the book, parents and psychosocial workers named the adaptive coping strategies, which were considered relevant, accessible to all and easy to understand.

“*These are good strategies that are the basis of what we're taught to promote mental health.”* (Evelyne, Mother of 11-year-old girl)

“*I don't usually let children know how I feel about a difficult situation because I think I'm protecting them. But this book helped me realise how important it is to communicate with them and how to do this.”* (Donovan, Father of a 7-year-old girl)

The taught strategies were also praised for being well illustrated, clearly explained for the 7- to 11-year-old age group and easily applicable in various situations. Because strategies were appropriate to a given context, the participants felt children could more easily identify the type of situation calling for the tools presented. Two psychosocial workers also commented on the diversity of the proposed learning methods (e.g., dance, drawings, sharing, games, etc.), which meant more children could be reached based on their learning styles and interests. Judith (psychosocial worker) specified: “*Excellent strategies, varied and accessible to all. They are well illustrated and explained for all ages.”*. One psychosocial worker, on the other hand, felt that strategies should not be presented as instructions, since this interrupted the storyline and could cause children to lose interest: “*At times, I didn't feel like I was reading a children's story anymore, but a document explaining a strategy*.” (Valerie, psychosocial worker)

The majority of children stated they had used or were thinking of using a strategy from the book in the near future. Among the strategies they had used or were considering using in the near future, the breathing technique was named most often. Half the children mentioned communication techniques (e.g., talking about their emotions, using “*I*” instead of “*you*”, looking at people when speaking). Also, Angelique (age 7) responded: “*Asking someone for help when you need it. I used this technique at school*.” Additionally, some parents intended to use, or reported having used, some of the strategies presented, as evidenced in the following:

“*We've already experimented them, and it has helped us during conflicts*.” (Jade, Mother of an 8-year-old girl and an 11-year-old boy);

“*They're great [strategies] and my kids are already using them.”* (Mathieu, Father of a 9-year-old girl and an 8-year-old boy).

At the same time, parents and children identified the strategies they perceived as particularly useful and helpful. Some named self-regulation strategies (e.g., self-soothing or self-calming), others the belly-breathing technique or the tips for sharing feelings (e.g., the importance of and ways to facilitate communication about feelings) and promoting effective communication, including “*speaking in the first person*”. The following comments reflect the strategies participants found helpful:

“*Talking about how you feel because it helps a lot*.” (Lea, age 11) “*The way we communicate. We lived for several years with a dad who blamed us for his bad behaviour, so the kids see from the book that that this isn't a good way to express themselves*.” (Layla, Mother of two boys, ages 8 and 10)

For the father of a 7-year-old girl, the problem-solving technique was the most relevant: “*As long as we're alive, we'll have problems. I think that mastering this technique early on will better prepare children for life*.”

#### Complementary Activities

All respondents described the activities as relevant, fun and interesting. Some parents and psychosocial workers emphasized that the activities improved the children's understanding of the story, allowed them to integrate the book's content, and made the story more interactive and dynamic.

“*There were fun games. (..) I hope you write more books like this because it explains to people how to relax and gives them activities to do when they're bored*.” (Rose-Anne, age 8)

“*The games were fun and helped us get to know each other better, develop new strategies and laugh together*.” (Jade, Mother of an 8-year-old girl and an 11-year-old boy)

Conversely, one father criticized the complementary activities as the least interesting aspect of the book: “*I liked the rest of the book less, even though it contains important information. I lost interest in reading after chapter 6. I found this part much more theoretical*.” Accordingly, he suggested the activities be separate from the book.

According to three psychosocial workers, Captain's reading comprehension questions are important because they allow for consolidating transmitted information, reinforcing what has been learned, and revisiting certain passages of the story with the children; as well, they make the reading experience more interactive and playful. These workers also recommend adding specific questions regarding the characters' reactions or general questions about problems experienced and ways to avoid certain situations or normalize the behaviors of persons with a mental illness instead of focusing solely on the tips and solutions in the questions asked. One worker, however, indicated this learning modality might not be of interest to all children, as it could prolong the reading.

In general, most child participants said they very much enjoyed the body percussion activities in the section *Pirate-Active*, and the psychosocial workers stated they were an interesting addition to the book. Only one child and one psychosocial worker reported they liked these activities least. Two workers said the activities allowed children to move and become aware of their physical sensations, making the book more dynamic. Only one mentioned she was not convinced of the activities' relevance or children's participation. Some psychosocial workers observed that the activities involved several persons (e.g., a few children together, a sibling), whereas a child might not have access to several persons willing to participate. Alternatives were therefore proposed to be added to the book, for example, a breathing or communication activity.

Furthermore, almost every child reported they enjoyed the pirates' emotional tools—including the pirate's mood thermometer—provided in this section. Only one said he liked them more or less. Parents, for their part, said they liked the way the book dealt with emotions and offered tips on attending to emotions and learning to regulate them. A father of a 11-year-old girl explained: “*I liked that it talks about emotions and gives tips on how to learn to work on those emotions.”*

Most of the psychosocial workers found these tools interesting and relevant, notably, because they provided children with visual tools to recognize and identify their moods and emotions. Florence specified: “*I think this activity is highly relevant since it puts a visual image on how the child is feeling.”* A few of them stated that they would have liked to see other features in this section (e.g., the breathing technique, a greater variety of emotions, questions allowing the child to draw parallels between the challenges experienced by the characters and their own life).

Overall, the majority of children reported they greatly enjoyed the tips and tools for sharing and regulating emotions presented in the section *Tips from Diane La Sage*, while psychosocial workers perceived this section as interesting, relevant and instructive. The reinvestment of the tools presented in the book and their connection with the child's experiential life were two more positives aspects mentioned. Sophie mentioned: “*It's good to have activities that refer to the child; it allows children to transfer elements of the story into their own life.”*.

One psychosocial worker especially appreciated the first tip (*calm your emotional storm*) because it offered children various ways to regulate their emotions, allowing each to choose the solution best suited to a particular situation. However, one psychosocial worker suggested an activity be included motivating children to reflect on the presence of some of the characters' behaviors in people in their own lives (e.g., the captain's refusal to get out of bed or Louis Le Curieux's anxiety).

Besides, most child participants reported they greatly appreciated the quiz (*Which of the pirates are you most like?*). Psychosocial workers considered it to be playful, interactive and interesting for opening discussion and encouraging introspection (e.g., identifying personal strengths). However, one proposed the following change: “*It's pretty obvious what answer they have to give to look like the pirate of their choice. A simple key might make identification of the pirate less obvious*.”

Most of the children also greatly enjoyed the cooking activities (*Jeanne La Borgne's special recipes*) aimed at promoting quality time between parents and children; only one child reported having little interest. Some psychosocial workers believed these activities could help children develop skills, self-confidence, and good life habits; they reported that cooking was an interesting and fun way to encourage special parent-child moments. One person in particular commented: “*Nice idea, well presented with “pirate” ingredients. A nice activity that's not often suggested to children and their parents*.” (Marianne, psychosocial worker). Conversely, one psychosocial worker did not find these activities necessary for understanding the book and the tools, while another questioned the relevance of the recipes. A few suggested that changes be made (e.g., use simpler and inexpensive ingredients).

In addition, children report enjoying the *My pirate team* activity, which focuses on identifying their support network. The psychosocial workers also approved this activity because the support network is “*super important to focus on*”. In general, they appreciated parental involvement in this activity, as most mentioned it was an excellent idea.

Then, child participants were highly enthusiastic about the drawing activity in *Pirate apprentices' corner* aimed at promoting children's self-esteem and reinforcing the dynamic aspect of the book. Only one child reported being uninterested. Two psychosocial workers considered this section relevant, but non-essential. Psychosocial workers, in fact, felt that drawing had several possible benefits for children because it fostered a sense of pride and the integration of content and encouraged children's participation.

### Content Readability and Adaptation to Child's Age

From parents' perspectives, the strategies presented allowed children to understand how to find solutions without pushing them too hard. Josiane, a mother of a 9 year old girl and an 8 year old boy stated: “*It's easy to understand and fun at the same time*.” In this regard, psychosocial workers believe children should be able to understand the information conveyed in the book. They reported that the book was, on the whole, easy to read and understand thanks to simple words, clear and playful explanations and a good number of illustrations. Judith, a psychosocial worker, explained: “*The book is still quite a long read, but I think the chapters and interactive aspect should hold children's interest and keep parents involved as well*.”

Nevertheless, a minority of parents and psychosocial workers stated that some of the terminology used could be difficult to understand, especially for younger children. In this regard, some psychosocial workers reported that the children may need support to understand certain concepts presented in the book (e.g., the usefulness and origin of emotions) and to do the activities (e.g., body percussion activities), despite the fact that the book is generally easy to read. This limitation was noted by a mother who participated in the study with her 9-year-old son: “*There were a few words I had to explain to him, but generally he understood the story very well. He has a lot of trouble with reading comprehension, so I had to help him make the connections with real life because he wasn't making the connections himself*.”

Overall, only, one parent and one psychosocial worker suggested that some activities may be less adapted to a child's age (e.g., special recipes section) or gender (e.g., dance activities). Furthermore, two psychosocial workers expressed concerns about the book's readability and adaptation for school-aged children. Among other things, they found that the story was somewhat “childish” considering the target audience (particularly for 11-year-old children) and did not sufficiently consider the preferences of all school-aged children. With this in mind, one worker suggested that a similar bibliotherapy be created with a different theme (e.g., sports). This way, some children “*might get a little more out of the story*”. However, she believed there was little chance a child would fail to enjoy this type of reading.

## Discussion

The purpose of the present study was to conduct a formative evaluation of an IPT-based book (*Le Trésor de l'Île Rouge)* targeting COPMI. The evaluation documents the perspective of families with parental mental illness and of psychosocial workers toward the acceptability, appropriateness and utility of the bibliotherapy. Results suggest the book is useful and well-adapted for 7- to 11-year-old children with a mentally ill parent and their families.

First, the results of our study support the findings of previous research indicating that bibliotherapy such as the one evaluated in the current study may help children learn concrete strategies and tools to cope with the personal and familial challenges they face on a daily basis (9). Children are introduced to different coping strategies and tools for dealing with adversity thanks to the adventures and challenges faced by the characters in the story. Through bibliotherapy, children can identify with characters and situations, create connections to their experiences and discover new and healthy adaptive coping strategies to use in everyday life (53, 54, 58). In this regard, almost all parents and children in this study reported they had used or intended to use a strategy taught in the book. Also, all participating psychosocial workers underlined they would use the book in their practice and recommend it to COPMI and their families.

Second, results show the book's use of a story and dynamic activities is relevant for helping families initiate discussions on sensitive subjects like parental mental health, a finding consistent with the recognized effects of bibliotherapy (52). Indeed, bibliotherapy is frequently used in interventions to help create a safe space for dialogue with children, facilitate discussion of sensitive and emotional content, and encourage reflection and exploration of children's experiences (53). Furthermore, the parents in our study reported they particularly appreciated the parallels between situations in the book and those in everyday life. The literature as well identifies this as a benefit of using bibliotherapy (52–54, 58). Indeed, stories can depict real aspects of family and community life for children, thereby strengthening family relationships (57).

Third, participants say they appreciate the various, concrete and simple coping strategies (e.g., tips for social-emotional regulation and problem-solving) offered throughout the book. They view the strategies proposed as relevant for helping school-aged COPMI respond to challenges (e.g., family conflicts, communication problems, stress, feelings of isolation). They recognize that learning these strategies may help foster better interpersonal relationships, particularly regarding parents and family members, and improve their social support network and well-being. This further supports the relevance of the recommendations for targeting these psychosocial factors in preventive interventions for families with parental mental illness (29, 30, 60).

### Clinical Implications

Results on the whole underline the relevance and acceptability of the book *Le Trésor de l'Île Rouge* for elementary school-aged COPMI and their families. This high-risk context where many potential consequences can negatively impact a child's well-being (1) necessitates the development and evaluation of tools and psychoeducational interventions to prevent mental health problems or psychological distress in these children. To our knowledge, this is the first formative evaluation of a bibliotherapy targeting school-aged COPMI. In light of the results of the present study, it may be viewed as a promising psychoeducational prevention tool.

Besides, some elementary school-aged children living with parental mental illness already present symptoms of mental illness or experience significant distress. These children are in dire need of psychosocial help and support. Many parents who have a mental illness may worry about their children's mental health and look for tools or psychosocial support services to help them cope (37). These families may face important additional challenges owing to the current pandemic and the limited capacity of mental health services to meet their needs (38, 39); such challenges can include obstacles to accessing care and services (e.g., long waiting lists and the absence of dedicated care pathways and intervention tools adapted to their needs and family issues) (40). In this context, bibliotherapy like the one in this study could offer a useful form of support for children and families who are waiting for psychosocial services or hesitate to accept help from a professional.

Moreover, according to some authors (52), bibliotherapy can also be an effective universal prevention tool for promoting mental health in primary school children. The results of the present study underscore the relevance of combining storytelling and activities adapted to the developmental level of primary school children. The book captures their attention and facilitates learning. The use of tools and adapted activities can promote children's understanding of content, facilitate discussions on sensitive issues, normalize their experiences and reinforce their interest (42, 52). Therefore, a book like *Le Trésor de l'Île Rouge* may help parents, psychosocial workers and teachers to accompany children coping with difficult everyday situations and allow them to recognize and fulfill their potential. The book can be used in a variety of ways: individually by the child, together as a family (parent-child or sibling), or in the classroom (teacher-led individual or group educational activity).

Overall, results support the importance of adapting tools and interventions to the specific age group and developmental level of targeted children, as many researchers suggest (42, 51). More broadly, the involvement of children themselves in the evaluation of interventions ensures the evaluations are accessible, relevant and appreciated, which then ensures that the services made available to them are actually used (50). Results show that the book was favorably viewed and well-appreciated by families and psychosocial workers, who also praised the book's format, illustrations and characters. According to participants, the content is clearly written, playful, well-illustrated and easy to understand; child participants were able to understand and retain the information conveyed. It could be assumed the book's readability and suitability for elementary school-aged children influenced their appreciation of it. Yet the children's obvious enjoyment proves that it was, in fact, a good choice for them. As stated above, the use of tools and adapted activities can promote children's understanding of content, facilitate discussions on sensitive topics, normalize their experiences and reinforce their interest, according to some authors (42). In consequence and considering that parents also appear to appreciate this book, it's possible children and their parents will be more inclined to use it on a daily basis, for example, to spend time together as a family or use the tools included in the book. Because there is often a high attrition rate in interventions targeting families with parental mental illness and maintaining engagement is a well-known challenge (21), offering an adapted bibliotherapy as an intervention modality could help increase the retention and participation of children and families. Ultimately, an intervention that aligns with the characteristics of the targeted clientele and responds to their needs makes for a smoother implementation process (48) and ensures that the services made available are actually used (49).

### Strengths and Limitations of the Study

To our knowledge, this study is the first of its kind to validate the acceptability, appropriateness and utility of a bibliotherapy for COPMI. Generally speaking, the effects of preventive interventions are usually evaluated without considering their implementation or first documenting their acceptability, adaptation or satisfaction by those concerned (47). In the current study, data were collected and analyzed from three different types of respondents (children, parents and psychosocial workers), therefore offering different perspectives and increasing results validity (71).

Additionally, an important strength of the study lies in that scarce empirical data are available regarding the satisfaction of children in this particular age group living with parental mental illness. Children's views of the content and modalities proposed in intervention programs developed to support them are rarely collected, despite the importance (72) and numerous benefits (73) of doing so. Their views may often differ from those of parents or psychosocial workers (44). Data from research using this type of design are known to be more valid and accurate because they come from the children themselves, the primary target audience (73, 74). Children are in the best position to share their perspectives (ideas, opinions, suggestions) and experiences (75, 76). This formative evaluation contributed to the development of a bibliotherapy adapted to children's preferences, needs and developmental level.

The project also has certain limitations. First, the small number of participants limits generalisability of results. Further evaluation with a larger number of participants is therefore recommended to verify whether similar results are obtained when the book is offered to larger samples and more diverse clienteles. This broader study would ensure representativeness of results and increase the potential for generalization. Second, variables were measured using measurement instruments designed by the research team, which nevertheless met the research objectives. Finally, certain biases may have interfered with the validity of the results obtained, such as participants' reactivity (e.g., trying to please, avoiding awkward responses or criticism) or participants' expectations (e.g., social desirability), particularly among children (73).

## Conclusion

In conclusion, this research has documented the satisfaction of children, parents and psychosocial workers with regard to a bibliotherapy targeting elementary school-aged COPMI. Overall, results show that families and psychosocial workers had a favorable opinion of the book, perceiving it to be relevant, useful and helpful. Thus, it appears well adapted to the developmental level of school-aged children. This research highlights the importance of adapting the content and modalities of interventions to children's interests, preferences, needs and developmental level. Future research should focus on promoting children's participation in the research process. It's important to develop a research culture where children's voices are heard and their involvement in the activities and decisions affecting them is an integral part of everyday research practice. Furthermore, psychosocial workers should be made aware that children, even school-aged children, require adaptive strategies to cope with the stressors they encounter. Bibliotherapy resources such as *Le Trésor de l'Île Rouge* could be made available in settings frequented by children and parents, including schools and community organizations in a perspective of universal prevention. Such resources could also be offered to parents with a mental disorder who are eager to find tools supporting family resilience.

## Data Availability Statement

The raw data supporting the conclusions of this article will be made available by the authors, without undue reservations. Further inquiries should be sent to the corresponding author.

## Ethics Statement

The studies involving human participants were reviewed and approved by Ethics Committee for Research Involving Humans, University of Quebec in Outaouais, Saint-Jérôme (Québec), Canada. Written informed consent to participate in this study was provided by the participants' legal guardian/next of kin.

## Author Contributions

KV and GP undertook and led the development of the research design and methodology, with contributions from AV. KV and GP undertook the literature review and led the data collection, with contributions from AV. KV led the data analysis and interpretation of findings, with contributions from GP and AV. KV and GP wrote the manuscript with editing/contributions from AV. All authors contributed to the article and approved the submitted version.

## Conflict of Interest

The authors declare that the research was conducted in the absence of any commercial or financial relationships that could be construed as a potential conflict of interest.

## Publisher's Note

All claims expressed in this article are solely those of the authors and do not necessarily represent those of their affiliated organizations, or those of the publisher, the editors and the reviewers. Any product that may be evaluated in this article, or claim that may be made by its manufacturer, is not guaranteed or endorsed by the publisher.

## References

[1] Reupert AE. J Maybery D Kowalenko NM. Children whose parents have a mental illness: prevalence, need and treatment. Med J Aust. (2012) 1:7–9. 10.5694/mjao11.11200 25369850

[2] Association AP. Diagnostic and Statistical Manual of Mental Disorders. 5ed. Arlington: Elsevier-Masson (2015).

[3] Brockington I Chandra P Dubowitz H Jones D Moussa S Nakku J. WPA guidance on the protection and promotion of mental health in children of persons with severe mental disorders. World psychiatry: official journal of the World Psychiatric Association (WPA). (2011) 10:93–102. 10.1002/j.2051-5545.2011.tb00023.x 21633678 PMC3104877

[4] Sell M Daubmann A Zapf H Adema B Busmann M Stiawa M. Family functioning in families affected by parental mental illness: parent, child, and clinician ratings. Int J Environ Res Public Health. (2021) 18:79–85. 10.3390/ijerph18157985 34360277 PMC8345719

[5] Beardslee W Gladstone T O'Conner E. Transmission and prevention of mood disorders among children of affectively parents: a review. J Am Acad Child Adolesc Psychiatry. (2011) 50:1098–109. 10.1016/j.jaac.2011.07.020 22023998

[6] Gladstone TRD Beardslee WR Diehl A. The impact of parental depression on children. In: Reupert A, Maybery D, Nicholson J, Gopert M, Seeman MV, editors. Parental Psychiatric Disorder: Distressed Parents and Their Families. Cambridge University Press (2015). p. 117–26. 10.1017/CBO9781107707559.013

[7] Aldrige J Becker S. Children Caring for Parents With Mental Illness: Perspectives of Young Carers, Parents and Professionals. Bristol: The Policy Press (2003).

[8] Cooklin A. Promoting children's resilience to parental mental illness: engaging the child's thinking. Adv Psychiatr Treat. (2013) 19:229–40. 10.1192/apt.bp.111.009050

[9] Riebschleger J Costello S Cavanaugh DL Grové C. Mental health literacy of youth that have a family member with a mental illness: outcomes from a new program and scale. Front Psychiatry. (2019) 10:2. 10.3389/fpsyt.2019.00002 30778305 PMC6369184

[10] Reupert A Gladstone B Helena Hine R Yates S McGaw V Charles G. Stigma in relation to families living with parental mental illness: an integrative review. Int J Ment Health Nurs. (2021) 30:6–26. 10.1111/inm.12820 33283387

[11] Tabak I Zablocka-Zytka L Ryan P Poma SZ Joronen K Vigano G. Needs, expectations and consequences for children growing up in a family where the parent has a mental illness. Int J Ment Health Nurs. (2016) 25:319–29. 10.1111/inm.12194 27278508

[12] Hosman CM van Doesum KT van Santvoort F. Prevention of emotional problems and psychiatric risks in children of parents with a mental illness in the Netherlands: I. the scientific basis to a comprehensive approach. AeJAMH. (2009) 8:250–63. 10.5172/jamh.8.3.250

[13] Bell M Bayliss DM Glauert R Harrison A Ohan J. Children of parents who have been hospitalised with psychiatric disorders are at risk of poor school readiness. Epidemiol Psychiatr Sci. (2019) 28:508–20. 10.1017/S2045796018000148 29633682 PMC6998916

[14] Goodman SH Rouse MH Connell AM Broth MR Hall CM Heyward D. Maternal depression and child psychopathology: a meta-analytic review. Clin Child Fam Psychol Rev. (2011) 14:1–27. 10.1007/s10567-010-0080-1 21052833

[15] Dean K Stevens H Mortensen PB Murray RM Walsh E Pedersen CB. Full spectrum of psychiatric outcomes among offspring with parental history of mental disorder. Arch Gen Psychiatry. (2010) 67:822–9. 10.1001/archgenpsychiatry.2010.86 20679590

[16] Siegenthaler E Munder T Egger M. Effect of preventive interventions in mentally ill parents on the mental health of the offspring: systematic review and meta-analysis. J Am Acad Child Adolesc Psychiatry. (2012) 51:8–17. 10.1016/j.jaac.2011.10.018 22176935

[17] Christiansen H Anding J Schrott B Rohrle B. Children of mentally ill parents-a pilot study of a group intervention program. Front Psychol. (2015) 6:1494. 10.3389/fpsyg.2015.01494 26539129 PMC4611090

[18] Kato N Yanagawa T Fujiwara T Morawska A. Prevalence of children's mental health problems and the effectiveness of population-level family interventions. J Epidemiol. (2015) 25:507–16. 10.2188/jea.JE20140198 26250791 PMC4517988

[19] Reupert AE Cuff R Drost L Foster K van Doesum KTM van Santvoort F. Intervention programs for children whose parents have a mental illness: a review. Med J Aust. (2012) 1:18–22. 10.5694/mjao11.11145 25369843

[20] Hodgins S Lane C Lapalme M LaRoche C Lavoie F Kratzer L. (editors). A Critical Review of the Literature of Children at Risk for Major Affective Disorders. Ottawa, ON: The Strategic Fund for Children's for Children's Mental Health (1994).

[21] van Doesum KT Hosman CM. Prevention of emotional problems and psychiatric risks in children of parents with a mental illness in the Netherlands: II Interventions. AeJAMH. (2009) 8:264–76. 10.5172/jamh.8.3.264

[22] Weissman MM Wickramaratne P Gameroff MJ Warner V Pilowsky D Kohad RG. Offspring of depressed parents: 30 years later. Am J Psychiatry. (2016) 173:1024–32. 10.1176/appi.ajp.2016.15101327 27113122

[23] Beardslee W Keller M Lavori P Staley J Sacks N. The impact of parental affective disorder on depression in offspring: a longitudinal follow-up in a nonreferred sample. J Am Acad Child Adolesc Psychiatry. (1993) 32:723–30. 10.1097/00004583-199307000-00004 8340291

[24] Piché G Bergeron L Cyr M. Transmission intergénérationnelle des troubles intériorisés: Modèles théoriques et recherches empiriques. Can Psychol. (2008) 49:309–22. 10.1037/a0013995

[25] Drost LM van der Krieke L Sytema S Schippers GM. Self-expressed strengths and resources of children of parents with a mental illness: A systematic review. Int J Ment Health Nurs. (2016) 25:102–15. 10.1111/inm.12176 26692281

[26] Foster K O'Brien L Korhonen T. Developing resilient children and families when parents have mental illness: a family-focused approach. Int J Ment Health Nurs. (2012) 21:3–11. 10.1111/j.1447-0349.2011.00754.x 21692961

[27] Gladstone BM Boydell KM Seeman MV McKeever PD. Children's experiences of parental mental illness: a literature review. Early Interv Psychiatry. (2011) 5:271–89. 10.1111/j.1751-7893.2011.00287.x 21883973

[28] Lewandowski RE Verdeli H Wickramaratne P Warner V Mancini A Weissman M. Predictors of positive outcomes in offspring of depressed parents and non-depressed parents across 20 years. J Child Fam Stud. (2014) 23:800–11. 10.1007/s10826-013-9732-3 25374449 PMC4217704

[29] Marston N Stavnes K Van Loon LMA Drost LM Maybery D Mosek A. A content analysis of Intervention Key Elements and Assessments (IKEA): what's in the black box in the interventions directed to families where a parent has a mental illness? Child Youth Serv. (2016) 37:112–28. 10.1080/0145935X.2016.1104041

[30] Tapias E Coromina M Grases N Ochoa S. Psychological treatments with children of parents with mental illness: a systematic review. Child Youth Care Forum. (2021) 50:1107–30. 10.1007/s10566-021-09608-2

[31] Bettis AH Forehand R Sterba SK Preacher KJ Compas BE. Anxiety and depression in children of depressed parents: dynamics of change in a preventive intervention. J Clin Child Adolesc Psychol. (2018) 47:581–94. 10.1080/15374416.2016.1225503 27768384 PMC5459662

[32] Zalewski M Goodman SH Cole PM McLaughlin KA. Clinical considerations when treating adults who are parents. Clin Psychol. (2017) 24:370–88. 10.1111/cpsp.12209 29515293 PMC5836549

[33] Piché G Villatte A Bourque S. Trouble mental chez le parent : Enjeux familiaux et implications cliniques. Quebec: Presses de l'Université Laval (2021).

[34] Niemelä M Kallunki H Jokinen J Räsänen S Ala-Aho B Hakko H. Collective impact on prevention: let's talk about children service model and decrease in referrals to child protection services. Front Psychiatry. (2019) 10:64–70. 10.3389/fpsyt.2019.00064 30833911 PMC6387903

[35] van Santvoort F Hosman CM van Doesum KT Janssens JM. Effectiveness of preventive support groups for children of mentally ill or addicted parents: a randomized controlled trial. Eur Child Adolesc Psychiatry. (2014) 23:473–84. 10.1007/s00787-013-0476-9 24072523

[36] Grove C Reupert A Maybery D. Gaining knowledge about parental mental illness: how does it empower children? Child Fam Soc Work. (2015) 20:377–86. 10.1111/cfs.12086

[37] Afzelius M Plantin L Östman M. Families living with parental mental illness and their experiences of family interventions. J Psychiatr Ment Health Nurs. (2018) 25:69–77. 10.1111/jpm.12433 28906576

[38] Gagné M-H Piché G Clément M-È Villatte A. Families in confinement: a pre–post COVID-19 study. Couple Fam Psychol: Res and Pract. (2021). 10.1037/cfp0000179

[39] Furlong M McGilloway S Mulligan C Killion MG McGarr S Grant A. Covid-19 and families with parental mental illness: crisis and opportunity. Front Psychiatry. (2021) 12. 10.3389/fpsyt.2021.567447 34385936 PMC8353101

[40] Maybery D Reupert A. Parental mental illness: a review of barriers and issues for working with families and children. J Psychiatr Ment Health Nurs. (2009) 16:784–91. 10.1111/j.1365-2850.2009.01456.x 19824972

[41] Thanhäuser M Lemmer G de Girolamo G Christiansen H. Do preventive interventions for children of mentally ill parents work? results of a systematic review and meta-analysis. Curr Opin Psychiatry. (2017) 30:283–99. 10.1097/YCO.0000000000000342 28505032

[42] Garber J Frankel SA Herrington CG. Developmental demands of cognitive behavioral therapy for depression in children and adolescents: cognitive, social, and emotional processes. Annu Rev Clin Psychol. (2016) 12:181–216. 10.1146/annurev-clinpsy-032814-112836 27019397 PMC5441981

[43] Van Doesum K Maia T Pereira C Loureiro M Marau J Toscano L. The impact of the “Semente” program on the family-focused practice of mental health professionals in Portugal. Front Psychiatry. (2019) 10:305. 10.3389/fpsyt.2019.00305 31133896 PMC6514231

[44] Grové C Reupert A Maybery D. The perspectives of young people of parents with a mental illness regarding preferred interventions and supports. J Child Fam Stud. (2016) 25:3056–65. 10.1007/s10826-016-0468-827782017

[45] Reupert A Goodyear M Eddy K Alliston C Mason P Maybery D. Australian programs and workforce initiatives for children and their families where a parent has a mental illness. AeJAMH. (2009) 8:277–85. 10.5172/jamh.8.3.277

[46] Craig P Ruggiero E Frohlich KL Mykhalovskiy E White M. Taking Account of Context in Population Health Intervention Research: Guidance for Producers, Users and Funders of Research: National Institute for Health Research (2018).

[47] Loechner J Starman K Galuschka K Tamm J Schulte-Korne G Rubel J. Preventing depression in the offspring of parents with depression: a systematic review and meta-analysis of randomized controlled trials. Clin Psychol Rev. (2018) 60:1–14. 10.1016/j.cpr.2017.11.009 29305152

[48] Chen HT. Practical Program Evaluation: Assessing and Improving Planning Implementation, and Effectiveness. London: Sage Publishing (2005).

[49] Collins E Clark H. Supporting Young People to Make Change Happen: A Review of Theories of Change. ActKnowledge and Oxfam Australia. (2013).

[50] Patton MQ. Developmental evaluation: Applying complexity concepts to enhance innovation and use. New York, NY: Guilford Press (2010).

[51] Newman L Birleson P. Mental health planning for children and youth: is it developmentally appropriate? Australas Psychiatry. (2012) 20:91–7. 10.1177/1039856211432479 22467561

[52] Mendel MR Harris J Carson N. Bringing Bibliotherapy for Children to Clinical Practice. J Am Acad Child Adolesc Psychiatry. (2016) 55:535–7. 10.1016/j.jaac.2016.05.008 27343878

[53] Gervais J Bouchard S Gagnier N. Using Stories to Prevent Anxiety Disorders in a School Context : Dominique's Handy Tricks Program. IntechOpen (2011).

[54] Goddard AT. Children's books for use in bibliotherapy. J Pediatr Health Care. (2011) 25:57–61. 10.1016/j.pedhc.2010.08.006 21147409

[55] Montgomery P Maunders K. The effectiveness of creative bibliotherapy for internalizing, externalizing, and prosocial behaviors in children: a systematic review. Child Youth Serv Rev. (2015) 55:37–47. 10.1016/j.childyouth.2015.05.010

[56] Moldovan R Cobeanu O David D. Cognitive bibliotherapy for mild depressive symptomatology: randomized clinical trial of efficacy and mechanisms of change. Clin Psychol Psychother. (2013) 20:482–93. 10.1002/cpp.1814 22941790

[57] Pulimeno M Piscitelli P Colazzo S. Children's literature to promote students' global development and wellbeing. Health Promot. Perspect. (2020) 10:13. 10.15171/hpp.2020.05 32104653 PMC7036210

[58] Heath MA Smith K Young EL. Using children's literature to strengthen social and emotional learning. Sch Psychol Int. (2017) 38:541–61. 10.1177/014303431771007032046001

[59] Tussing HL Valentine DP. Helping adolescents cope with the mental illness of a parent through bibliotherapy. Child Adolesc Soc Work J. (2001) 18:455–69. 10.1023/A:1012992116742

[60] Reupert AE Maybery D. “Knowledge is power”: educating children about their parent's mental illness. Soc Work Health Care. (2010) 49:630–46. 10.1080/00981380903364791 20711943

[61] Bearslee W Wright E Gladstone T Forbes P. Long-term effects from a randomized trial of two public health preventive interventions for parental depression. J Fam Psychol. (2007) 21:703–13. 10.1037/0893-3200.21.4.703 18179342

[62] Piche G Villatte A Habib R Vetri K Beardslee W. FAMILLE+: A Multifamily Group Program for Families with Parental Depression. J Parent Fam Mental Health. (2020) 5:1–4. 10.7191/parentandfamily.1014

[63] Piché G Vetri K Villatte A Habib R. Évaluation pilote d'un programme d'intervention préventive ciblant les familles comprenant un parent ayant vécu un trouble dépressif. Revue de Psychoéducation. (2022) (in press).

[64] Withnell N Murphy N. Family Interventions in Mental Health. New York, NY: McGraw-Hill Education (2012).

[65] Mufson L Dorta KP Moreau D Weissman MM. Interpersonal Psychotherapy for Depressed adolescents. 2nd ed. New York, NY: Guilford Press (2004).

[66] Young JF Kranzler A Gallop R Mufson L. Interpersonal psychotherapy-adolescent skills training: Effects on school and social functioning. School Ment Health. (2012) 4:254–64. 10.1007/s12310-012-9078-9 23393545 PMC3564646

[67] Dietz LJ. Family-based interpersonal psychotherapy: an intervention for preadolescent depression. Am J Psychother. (2020) 73:22–8. 10.1176/appi.psychotherapy.20190028 32050785

[68] Mahan RM Swan SA Macfie J. Interpersonal psychotherapy and mindfulness for treatment of major depression with anxious distress. Clin Case Stud. (2018) 17:104–19. 10.1177/153465011875653027541331

[69] Villatte A Piché G Benjamin S. Perceived support and sense of social belonging in young adults who have a parent with a mental illness. Front Psychiatry. (2022) 12:1–15. 10.3389/fpsyt.2021.793344 35095606 PMC8792737

[70] Vetri K. L'adaptation d'un dispositif de bibliothérapie pour les enfants âgés de 7 à 11 ans vivant avec un parent ayant un trouble mental. Saint-Jérôme, QC: Université du Québec en Outaouais (2022).

[71] Gibson CB. Elaboration, Generalization, Triangulation, and Interpretation. Organ Res Methods. (2016) 20:193–223. 10.1177/1094428116639133

[72] Bee P Bower P Byford S Churchill R Calam R Stallard P. The clinical effectiveness, cost-effectiveness and acceptability of community-based interventions aimed at improving or maintaining quality of life in children of parents with serious mental illness: a systematic review. Health Technol Assess. (2014) 18:100–250. 10.3310/hta18080 24502767 PMC4780907

[73] Kennan D Dolan P. Justifying children and young people's involvement in social research: assessing harm and benefit. Ir J Sociol. (2017) 25:297–314. 10.1177/0791603517711860

[74] Broström S. Children's participation in research. Int J Early Years Educ. (2012) 20:257–69. 10.1080/09669760.2012.715407

[75] Kellett M. Empowering children and young people as researchers: overcoming barriers and building capacity. Child Indic Res. (2011) 4:205–19. 10.1007/s12187-010-9103-1

[76] Nabors LA E-Libro C. Research Methods for Children. Hauppauge: Nova Science Publishers, Inc (2013).

[77] Brady B. Developing children's participation: lessons from a participatory IT project. Child Soc. (2006) 21:31–41. 10.1111/j.1099-0860.2006.00024.x18844548

[78] International Q. N'Vivo (2020).

[79] Thomas DR, A. general inductive approach for analyzing qualitative evaluation data. Am J Eval. (2006) 27:237–46. 10.1177/1098214005283748

